# Acoustic separation and concentration of exosomes for nucleotide detection: ASCENDx

**DOI:** 10.1126/sciadv.adm8597

**Published:** 2024-03-08

**Authors:** Ty D. Naquin, Aidan J. Canning, Yuyang Gu, Jianing Chen, Chloe M. Naquin, Jianping Xia, Brandon Lu, Shujie Yang, Aleksandra Koroza, Katherine Lin, Hsin-Neng Wang, William R. Jeck, Luke P. Lee, Tuan Vo-Dinh, Tony Jun Huang

**Affiliations:** ^1^ Department of Mechanical Engineering and Materials Science, Duke University, Durham, NC 27708, USA.; ^2^ Department of Biomedical Engineering, Duke University, Durham, NC 27708, USA.; ^3^ Department of Pathology, Duke University Medical Center, Durham, NC 27708, USA.; ^4^ Harvard Medical School, Harvard University; Renal Division and Division of Engineering in Medicine, Department of Medicine, Brigham and Women’s Hospital, Boston, MA 02115, USA.; ^5^ Department of Bioengineering and Department of Electrical Engineering and Computer Science, University of California, Berkeley, Berkeley, CA 94720, USA.; ^6^ Institute of Quantum Biophysics, Department of Biophysics, Sungkyunkwan University, Suwon, Gyeonggi-do, Korea.; ^7^ Department of Chemistry and Nanoscience, Ewha Womans University, Seoul, Korea.

## Abstract

Efficient isolation and analysis of exosomal biomarkers hold transformative potential in biomedical applications. However, current methods are prone to contamination and require costly consumables, expensive equipment, and skilled personnel. Here, we introduce an innovative spaceship-like disc that allows Acoustic Separation and Concentration of Exosomes and Nucleotide Detection: ASCENDx. We created ASCENDx to use acoustically driven disc rotation on a spinning droplet to generate swift separation and concentration of exosomes from patient plasma samples. Integrated plasmonic nanostars on the ASCENDx disc enable label-free detection of enriched exosomes via surface-enhanced Raman scattering. Direct detection of circulating exosomal microRNA biomarkers from patient plasma samples by the ASCENDx platform facilitated a diagnostic assay for colorectal cancer with 95.8% sensitivity and 100% specificity. ASCENDx overcomes existing limitations in exosome-based molecular diagnostics and holds a powerful position for future biomedical research, precision medicine, and point-of-care medical diagnostics.

## Teaser

The ASCENDx platform integrates the isolation and detection of exosomal miRNA biomarkers for point-of-care applications.

## INTRODUCTION

Exosomes are cell-derived nanovesicles that have recently gained popularity as potential biomarkers for liquid biopsies due to their plethora of molecular cargo, such as nucleic acids and proteins (*1*–*5*). As a result, circulating exosomes are considered promising noninvasive biomarkers for investigating disease development, progression, and early disease detection (*6*–*9*). Exosome isolation, however, is challenging because of their small size and cohabitation with numerous other bioparticles in bodily fluids (*10*, *11*). Although several methods of separating exosomes have been developed, such as ultracentrifugation (*12*–*14*), polymer-assisted precipitation (*15*, *16*), and ultrafiltration (*17*), current techniques fail to meet research and clinical needs due to a lengthy processing time, large sample consumption, and limited purity and yield (*18*–*25*).

Clinical applications of exosomes are further hindered by limitations in biomarker analysis. Their lengthy procedures and cost-prohibitive reagent and instrumentation requirements limit conventional detection methods such as digital droplet polymerase chain reaction (PCR), benchtop reverse transcription quantitative PCR (RT-qPCR), sequencing, and microarray analysis (*26*–*29*). To circumvent these drawbacks, other sensing strategies have been implemented, including isothermal amplification (*30*, *31*), lateral flow tests (*32*), and electrochemical sensing assays (*33*), but they lack the specificity and sensitivity needed for widespread adoption in clinical settings due to the low concentrations of biomarkers in bodily fluids. Therefore, biomarker detection remains challenging without a unified exosome enrichment and in situ analysis platform to purify and concentrate biomarkers before detection.

Recently, microfluidics has emerged as a promising candidate for exosome separation and analysis (*34*–*36*). Current microfluidic technologies use a variety of mechanisms such as electric fields (*37*, *38*), magnetics (*39*–*41*), and acoustics (*42*–*51*) to control bioparticles and actuate fluids. However, existing microfluidic separation technologies have difficulties manipulating particles below 50 nm in size and removing the small, nonexosomal contaminants such as lipoproteins, leading to less reliable and consistent biomarker diagnosis. In addition, current technologies often produce relatively dilute samples and need help concentrating the exosomes, limiting the molecular information available for analysis. Furthermore, many existing microfluidic systems must be coupled with bulky analytical systems, making them unsuitable for point-of-care testing. For example, microfluidic separation often uses counting approaches such as nanoparticle tracking analysis (NTA) (*52*) and dynamic light scattering (*53*). Still, these technologies have bulky fingerprints and provide little diagnostic information. Conventional molecular assays such as Western blots and enzyme-linked immunosorbent assay (*54*) require large sample volumes, hampering their application in settings with large patient cohorts or limited specimen availability. Hence, there exists a need for rapid enrichment technology with integrated multiplexed biomarker sensing to bring the next-generation liquid biopsy analysis into fruition.

To overcome these limitations, we present our Acoustic Separation and Concentration of Exosomes for Nucleotide Detection (ASCENDx) platform, which allows the enrichment and detection of exosomes using a rotating microfluidic disc integrated with a multiplexed, plasmonic nanostar–based microRNA (miRNA) assay. We generate acoustofluidic disc rotation by coupling surface acoustic waves (SAWs) and the fluid layer on which the disc floats to enable centrifugation and fluid actuation within the microfluidic channels on the disc surface. We demonstrate various functionalities, such as the separation and concentration of both micro and nanoscale objects. The interplay between the centrifugal and drag forces leads to size- and density-dependent transport velocity, which we use here to separate the relatively large exosomes from protein contaminants in plasma. In addition, we functionalize a bimetallic nanostar substrate on the disc surface to verify our enrichment mechanism through label-free surface-enhanced Raman scattering (SERS) sensing of the concentrated exosome sample. Furthermore, we demonstrate our platform’s diagnostic potential through traditional RT-PCR and a multiplexed amplification–free SERS assay for detecting circulating colorectal cancer (CRC) miRNA biomarkers from patient plasma samples with high selectivity (95.8%) and specificity (100%).

## RESULTS

### Working mechanisms of ASCENDx

**Fig. 1.**
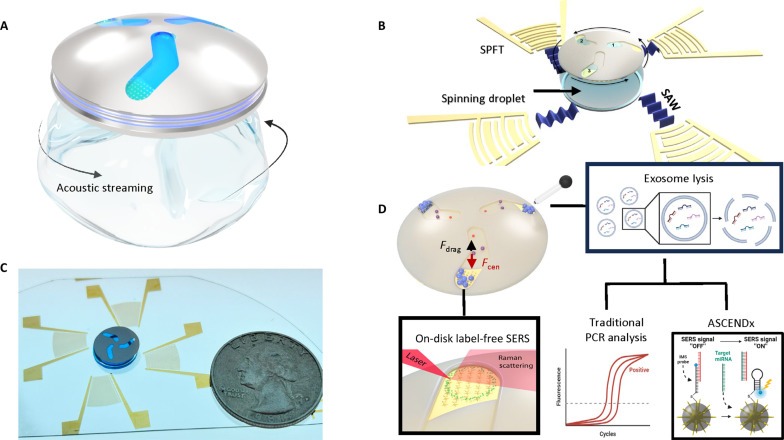
Operating mechanism of the ASCENDx platform. (**A**) Acoustic streaming within a water droplet deforms the liquid-air interface, causing the droplet to spin. When a disc is placed on top of the droplet, this streaming phenomenon causes the disc to rotate, leading to the concentration and separation of micro/nano objects. (**B**) Device photo and (**C**) schematic of the acoustofluidic disc unit of the ASCENDx platform. A water droplet is placed within a PDMS ring and situated between four SPFTs. A small disc with microfluidic structures patterned into its surface is placed atop the droplet. As SAWs propagate into the droplet, a helical vortex is formed, causing the droplet-disc system to rotate. (**D**) As the disc rotates, larger particles move to the ends of the channel first. In this region, we have immobilized bimetallic nanostars to facilitate SERS analysis of the concentrated sample. The enriched sample can be removed via pipette for PCR analysis and integration with our point-of-care miRNA assay. (D) was made with BioRender.com.

As shown in Fig. 1 (A to C), our ASCENDx platform consists of a droplet confined to a polydimethylsiloxane (PDMS) ring and two pairs of single-phase focused transducers (SPFTs) aligned along the flanks of the droplet. A small disc with slanted channels engraved on its surface sits atop the droplet. Four SAWs propagate into the droplet when an electrical signal is applied to the SPFTs, forming a circulatory acoustic streaming effect (*55*–*62*). As the input voltage increases, the droplet reaches a stable spinning state. The rotating droplet-disc system acts as a centrifuge when the acoustofluidic disc is placed on the droplet. As Fig. 1D illustrates, particles in the disc channels drift toward the disc edge, with larger particles migrating first. This phenomenon concentrates the large particles and creates a mechanism for separating the larger particles from the smaller ones that remain in the channel. Our unique disc design features a tilted channel geometry to enhance the enrichment further by shortening the path traveled by particles in the solution.

Differences in mass density cause phase separation between suspended particles in solution. This sedimentation of particles can be accelerated by applying a centrifugal force given by

$$F_{c}=r_{\text{avg}}\omega^{2}V_{p}∆\rho$$ (1)

where *r*_avg_ is the average radial distance of particles from the center of rotation, *V*_ρ_ is the volume of the particle, ω is the angular velocity, and ∆ρ is the difference between the density of the particle and the density of the medium. As particles of radius *r*_ρ_ migrate within a fluid of viscosity η, they experience a counteracting drag force proportional to the particle velocity

$$F_{\text{drag}}=6\pi \eta r_{\text{avg}}v$$ (2)

The interplay of the centrifugal force and the Stokes drag force determines the time required for complete sedimentation of particles of diameter 𝑑 across a channel width *L*

$$t_{d}=\frac{18\eta L}{\omega 2d^{2}\Delta \rho r_{\text{avg}}}$$ (3)

The commonly accepted vesicle density for exosomes is 1.19 g/cm^3^ with a medium viscosity of 1.55 cP (*63*). Using these values, a 5-mm-diameter disc spinning at 4000 rpm with 100-μm-wide channels will pellet 100% of 100-nm exosomes in just 31 min.

The shape and composition of plasmonic nanomaterials have been widely investigated for use in SERS sensing applications (*64*). The optical properties of these materials are attractive for integration into disease biomarker assays. To monitor exosome separation, the channel end of each acoustofluidic spinning disc has been functionalized with a plasmonic biosensing unit composed of bimetallic plasmonic nanostars with morphology optimized to generate a strong local electromagnetic field enhancement via localized surface plasmon resonance at 633 nm. This local electric field enhancement can be described where *E*_0_ is the magnitude of the incident electric field, 𝑔 is the field enhancement averaged over the surface of the particle, and *E*(ω) is the average magnitude of the electric field radiated by the particle at the incident frequency (*65*).

$$E\left(\omega \right)=gE_{0}$$ (4)

At a Stokes-shifted wavelength, there is a corresponding electric field enhancement factor *E*(ω′); however, the approximation *E*(ω′) = *E*(ω) is often made because the plasmon width is relatively large due to the Stokes shift. With the electric field enhancement factor, the SERS electromagnetic enhancement factor *EF*, which describes the intensity ratio between SERS and Raman scattering for a given molecule, is given by

EF=∣E(ω)∣4 (5)

By leveraging the local electromagnetic field enhancement generated by the plasmonic nanostar surface, we have established a SERS-based label-free exosome enrichment verification method on-disc, depicted in the lower left of Fig. 1D. The inverse Molecular Sentinel (iMS) assay is integrated within the plasmonic biosensing unit of the ASCENDx platform to detect CRC miRNA biomarkers contained within enriched exosome aliquots derived from patient plasma. The amplification-free assay (*66*, *67*) leverages a nonenzymatic DNA strand displacement process to trigger a conformational change of the probe and subsequently increase the SERS signal in the presence of target miRNA. Shown in the bottom right of Fig. 1D, the iMS probe is functionalized to the plasmonic nanostar surface and hybridized with a partially complementary placeholder strand. This hybridization complex results in a negligible SERS signal due to the distance created between the Raman dye and the plasmonic nanostar surface. The placeholder strand is displaced from the miRNA probe when in the presence of target miRNA, forming an entirely complementary duplex. Now free, the iMS probe self-hybridizes to form a hairpin structure. This conformational change brings the Raman dye within the area of high electric field enhancement along the plasmonic nanostar surface, resulting in a strong SERS signal capable of providing diagnostic information from patient tissue and plasma samples.

### Characterization of the acoustofluidic disc unit of ASCENDx

As SAWs enter the liquid, internal vortex streaming induces droplet rotation in the acoustofluidic disc unit of ASCENDx, shown in fig. S2. At low acoustic excitation, the droplet remains in its equilibrium shape due to the insufficient radiation pressure. As the excitation voltage increases, the droplet gradually experiences small vibrations until reaching a threshold value at which a stable spinning state is established, characterized by droplet deformation into an ellipsoidal shape with periodic rotational boundary oscillations. The region where the acoustic beam enters the droplet can substantially affect the droplet’s rotation speeds (*68*–*70*). To characterize and optimize the performance of the acoustofluidic disc unit of ASCENDx, we first investigated the effects of SPFT offset distance on droplet rotation. We performed numerical simulations to visualize the acoustic streaming pattern within the droplet and identify the ideal location for acoustic wave propagation by applying an acoustic beam on two flanks of the droplet (fig. S1). Our simulation results indicate that increasing SPFT offset distance maximizes the rotation speed due to the increased torque applied to the droplet axis. To validate our simulation results, we experimentally investigated the rotation speed of the droplet-disc system with variable transducer offsets. For a droplet with a radius of 5 mm, we found the optimal SPFT offset distance to be 3 mm, likely due to the spread of the acoustic beam not considered in our simulations and the additional SPFT pair included in the experiments (fig. S4).

**Fig. 2.**
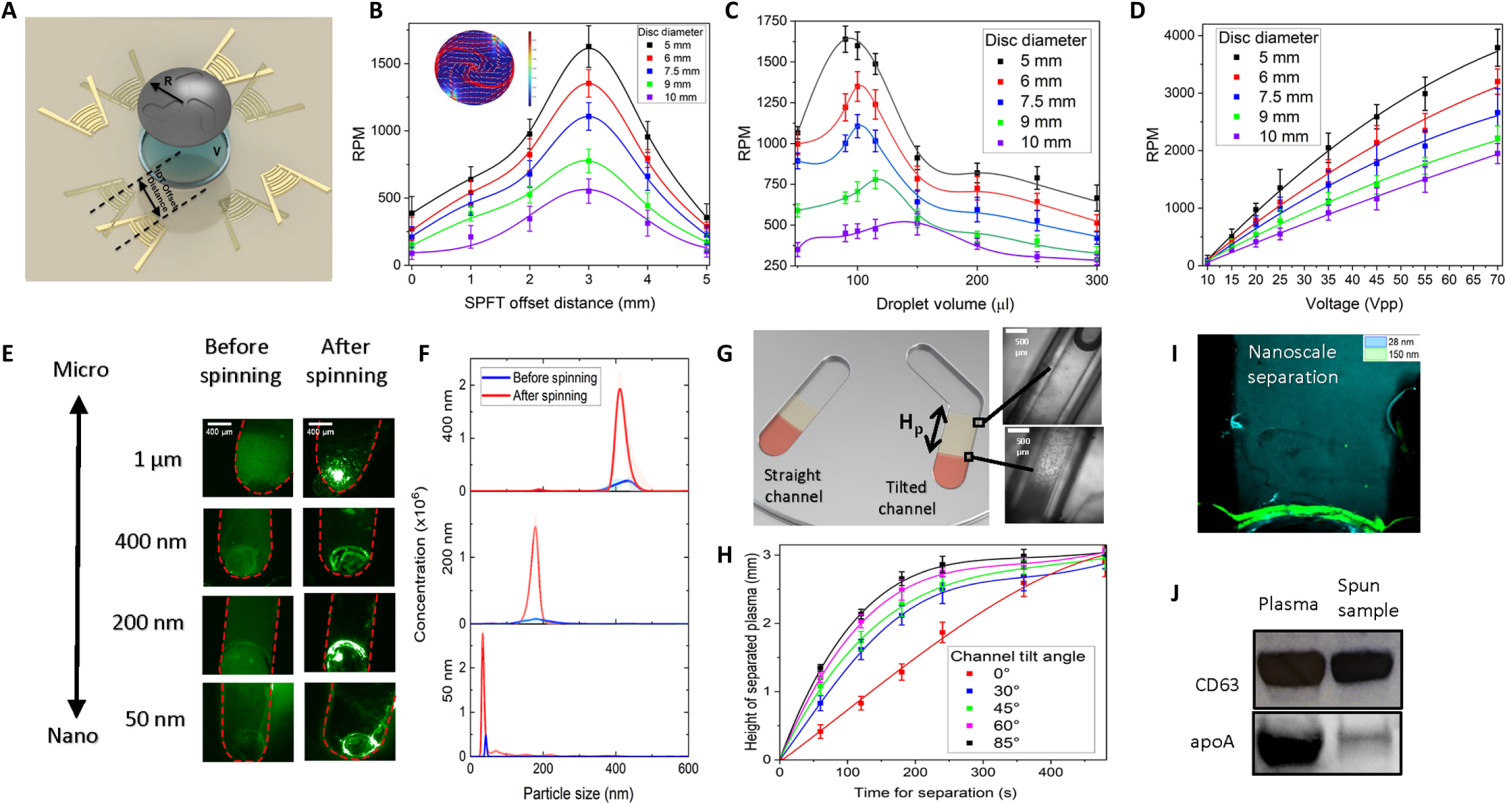
Characterization of the acoustofluidic disc unit of the ASCENDx platform. (**A**) Schematic defining the SPFT offset distance in relation to the droplet. Zero offset corresponds to the SPFT aligned directly with the center of the droplet. (**B**) Disc rotation speed with respect to SPFT offset distance. (**C**) Disc rotation speed with respect to droplet volume. (**D**) Disc rotation speed with respect to input voltage. (**E**) Particle concentration before and after acoustically driven disc spinning. (**F**) NTA results for nanoscale particles before and after acoustically driven disc spinning. (**G**) Illustration showing blood plasma separation in both straight and tilted channels. (**H**) Height of the plasma layer versus centrifugation time for various tilted channel geometries. Greater tilt angles expedite separation. (**I**) Overlay of microscope images showing the experimental result of particle separation with 150-nm (blue) and 28-nm (green) particles. Since larger particles concentrate first, by controlling the voltage and time applied, the 150-nm particles can be gathered to the channel edge, while the 28-nm particles remain dispersed throughout the channel. (**J**) Western blot analysis of the exosome biomarker CD63 and the protein biomarker Apolipoprotein A (apoA). Results demonstrate that exosomes remain present before and after acoustic processing and that apoA is present in a significantly lower degree after processing. This result suggests the separation of exosomes from proteins, resulting in a purer exosome sample.

Next, we explored the effect of droplet volume on the rotation efficiency of our platform, as shown in Fig. 2C. Our results indicate that, generally, lower droplet volumes produce greater rotational speeds. However, the disc size plays an important role here, as sufficient liquid must be present to hold the disc and maintain droplet integrity. In addition, the instability between the droplet and disc can induce pronounced precession of the disc, limiting the rotation speed. Therefore, there exists some optimal droplet volume corresponding to discs of various sizes to minimize precession and enable maximum centrifuge speeds (*59*, *71*, *72*). The viscosity of the liquid droplet is also an important consideration here. In this work, we use a water droplet, which experiences many reflections at the droplet interface. A more viscous liquid would attenuate the acoustic waves more and experience a lower streaming velocity (*73*). Last, we investigated the relationship between input power and revolution speeds, as shown in Fig. 2D. As expected, higher applied voltage generates increased gyration. The droplets used here experience negligible evaporation and thermal effects as voltage is increased because of their relatively large volume and enclosure by the PDMS ring and acoutstofluidic disc, depicted in fig. S5.

To showcase the enrichment capabilities of our ASCENDx device, we enclosed fluorescent polystyrene beads within the disc. As shown in Fig. 2E, the particles (50 nm, 200 nm, 400 nm, and 1 μm) are evenly dispersed throughout the channel before spinning but localized and heavily concentrated at the channel end after spinning, indicated by the significant changes in fluorescent intensity. The enrichment efficiency of the platform was quantified using NTA, shown in fig. S6, resulting in recovery rates of 94, 85, and 78% for 200-, 100-, and 50-nm particles, respectively. Our results (Fig. 2E) demonstrate that the ASCENDx platform can concentrate samples from micro- to nanoscales, making it ideally suited for a wide variety of applications in biology and medicine.

In addition to the concentration of homogenous solutions of particles, we also demonstrate differential separation based on size. The dynamics between particle dimensions, density, and the centrifugal force generate distinct migration speeds for different-sized particles within the channel. To highlight the microscale separation potential of our platform, we present the separation of plasma from whole blood. Ten microliters of blood was injected into the disc. As the disc spins, the larger blood cells experience greater centrifugal force, causing them to migrate to the channel end. As the height of the cell suspension decreases because of continued centrifugation, the height of the plasma layer *H*_ρ_ increases, creating a distinct interface between the cell suspension and plasma, as illustrated in Fig. 2G. Our device can isolate highly pure plasma samples from whole blood, as shown in the rightmost inset images. The resulting serum can be easily retrieved with a pipette or syringe for additional processing and analysis downstream.

Figure 2I presents the nanoscale separation of fluorescent particles. Here, we confine both 28- and 150-nm particles collectively in the channel. After spinning, the 150-nm particles relocate to the channel edge, while the 28-nm particles remain dispersed throughout the channel. Since the centrifugal force is proportional to the square of the particle size, the larger 150-nm particles experience significantly greater forces, causing them to concentrate well before the 28-nm particles, separating the two populations. Since exosomes range from 50 to 200 nm in size, this result highlights the potential for our device to separate exosomes from the many small (<10 nm) protein contaminants present in plasma.

We also confirm the separation of proteins from exosomes through Western blot analysis. CD63 is a common surface protein found on exosomes, while apolipoprotein A is a small free protein contained in plasma but not in exosomes. As shown in Fig. 2J, the original plasma sample contains high concentrations of both proteins, whereas the spun sample shows the presence of apolipoprotein A in lower concentrations while retaining the high CD63 content. Further validation of exosome yield and purity is demonstrated in fig. S8, which demonstrates a 120× increase in the purity of acoustically enriched samples with a yield of 86%.

### Characterization of the plasmonic biosensing unit of ASCENDx

**Fig. 3.**
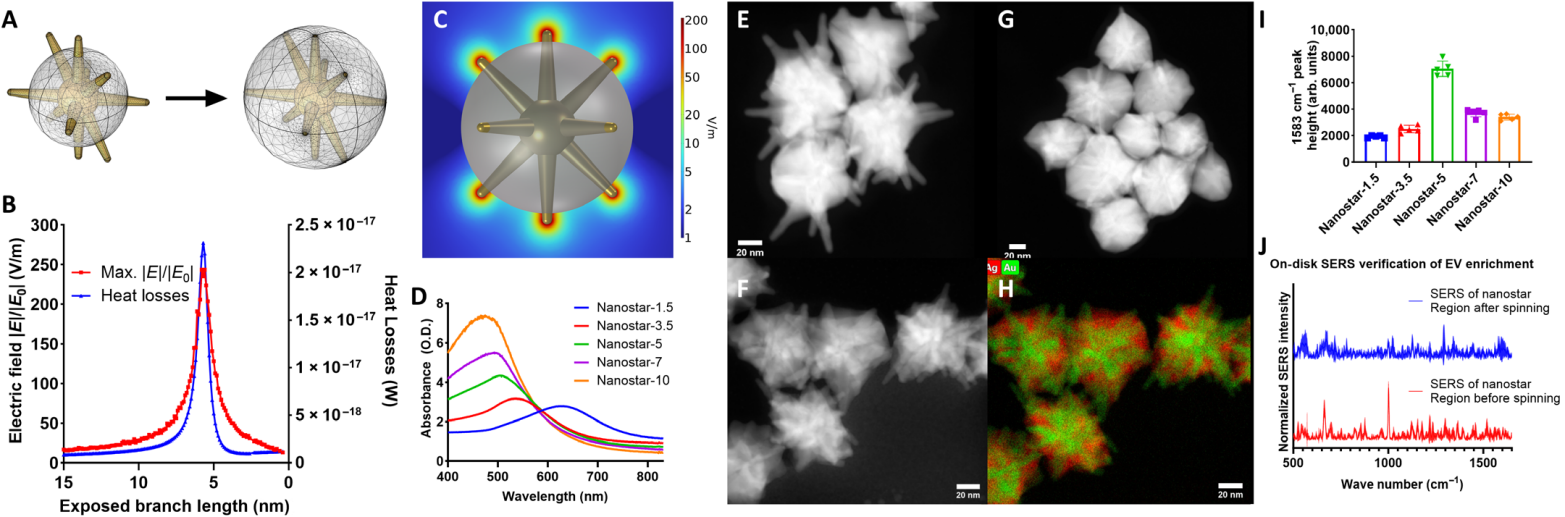
The plasmonic biosensing unit of ASCENDx. (**A**) Range of simulated plasmonic nanostar models. (**B**) Maximum electric field enhancement and heat loss of all simulated models. (**C**) Nanostar morphology with the greatest theoretical SERS enhancement factor. (**D**) Absorbance spectra of all nanoparticle morphologies synthesized. (**E** to **G**) High-angle annular dark-field (HAADF)–STEM images of nanostar-1.5, -5, and -10. (**H**) HAADF-STEM image of nanostar-5 particles. (**I**) SERS intensity at 1583 cm^−1^ of each different nanoparticle morphology. (**J**) SERS of plasmonic nanostar region on disc surface before and after spinning.

Plasmonic bimetallic nanostars are highly tunable sub–100-nm nanoparticles that produce intense local electromagnetic field enhancements to facilitate detection and sensing via SERS (*74*). Finite element modeling (FEM) of several bimetallic nanostar morphologies was performed with COMSOL Multiphysics 6.0 to determine the optimal nanostar morphology for disc integration. There, a gold nanostar model was constructed and held constant, while the silver layer thickness was varied. Figure 3A illustrates the range of silver thicknesses simulated in this study, 35 to 50 nm or 15 to 0 nm exposed branch length. The highest normalized electric field enhancement factor and total heat losses for each simulated model are shown in Fig. 3B. The heat loss values are obtained by integrating the resistive losses with the nanoparticle model volume and have been shown to be an accurate predictor of experimental SERS results (*75*). Figure 3C depicts the nanostar morphology and corresponding normalized electric field with the greatest theoretical SERS performance, with a maximum electric field enhancement factor of 235.16 V/m and a heat loss value of 2.3 × 10^−17^ W. This simulated nanostar had a silver layer thickness of 29.3 nm, or an exposed branch length of 5.7 nm. Overall, theoretical nanoparticle performance increases as the thickness of the silver layer increases until a maximum is reached, and then performance decreases as silver approaches the nanostar branch tips.

To verify the results of the FEM study, several different morphologies of bimetallic nanostars were synthesized. The formulation of the underlying gold nanostar was held constant, while the thickness of the silver coating was varied. The resulting morphologies are referred to here as nanostar-1.5, -3.5, -5, -7, and -10, with the value denoting the millimolar concentration of silver added to coat the internal gold nanostars. The increase in silver thickness results in blue shifting of the absorption spectra, as seen in Fig. 3D. Figure 3 (E to G) contains high-angle annular dark-field scanning transmission electron microscopy (STEM) images of representative nanostar-1.5, -5, and -10 particles. Silver is deposited on the exposed core of the nanostar and grows outward toward the branch tips. STEM–energy dispersive x-ray spectroscopy was used to examine the elemental structure of the nanostar-5 particles, which clearly reveals the underlying gold nanostar encased in a silver layer. To test how these morphological changes would affect the local electric field enhancement and resulting SERS signal intensity, *p*-mercaptobenzoic acid was added to all five nanostar morphologies. The SERS spectra of all samples were recorded, and the intensity of each spectrum at 1583 cm^−1^ is shown in Fig. 3I. As the silver layer thickness increases, the intensity of the SERS peak of interest increases, reaching a maximum with the nanostar-5 morphology, then decreasing as more silver is added to the particle surface, closely matching the results shown in Fig. 3B.

With the strong agreement between theoretical and experimental results, the nanostar-5 morphology was chosen as the nanoparticle type for incorporation onto the spinning acoustofluidic disc surface, as shown in the scanning electron microscopy image in fig. S9. The particles were incorporated into the end channel of the acoustofluidic disc to allow for on-disc label-free SERS verification of sample enrichment. Figure 3J shows averaged SERS spectra obtained from the sensing portion of the disc with and without sample spinning. In the stationary sample, several peaks emerge, most notably at 1002 cm^−1^, which has been shown to dominate the SERS spectrum of albumin and other globular proteins and is attributed to the symmetric stretching mode of phenylalanine (*76*). This peak was noticeably reduced in the spun sample, as well as peaks at 665, 875, 850, 1218^−1^, and 1176 cm^−1^, which all are assigned to globular plasma protein (*77*). The reduction in the SERS signal of plasma protein after acoustofluidic disc–based sample concentration is evidence of extracellular vesicle enrichment. As larger extracellular vesicles are concentrated on the edges of the acoustofluidic disc, steric hindrance will decrease the amount of globular protein that can interact with the nanostar surface and generate a subsequent SERS signal.

### Validation of the ASCENDx platform through RT-PCR using clinical samples

**Fig. 4.**
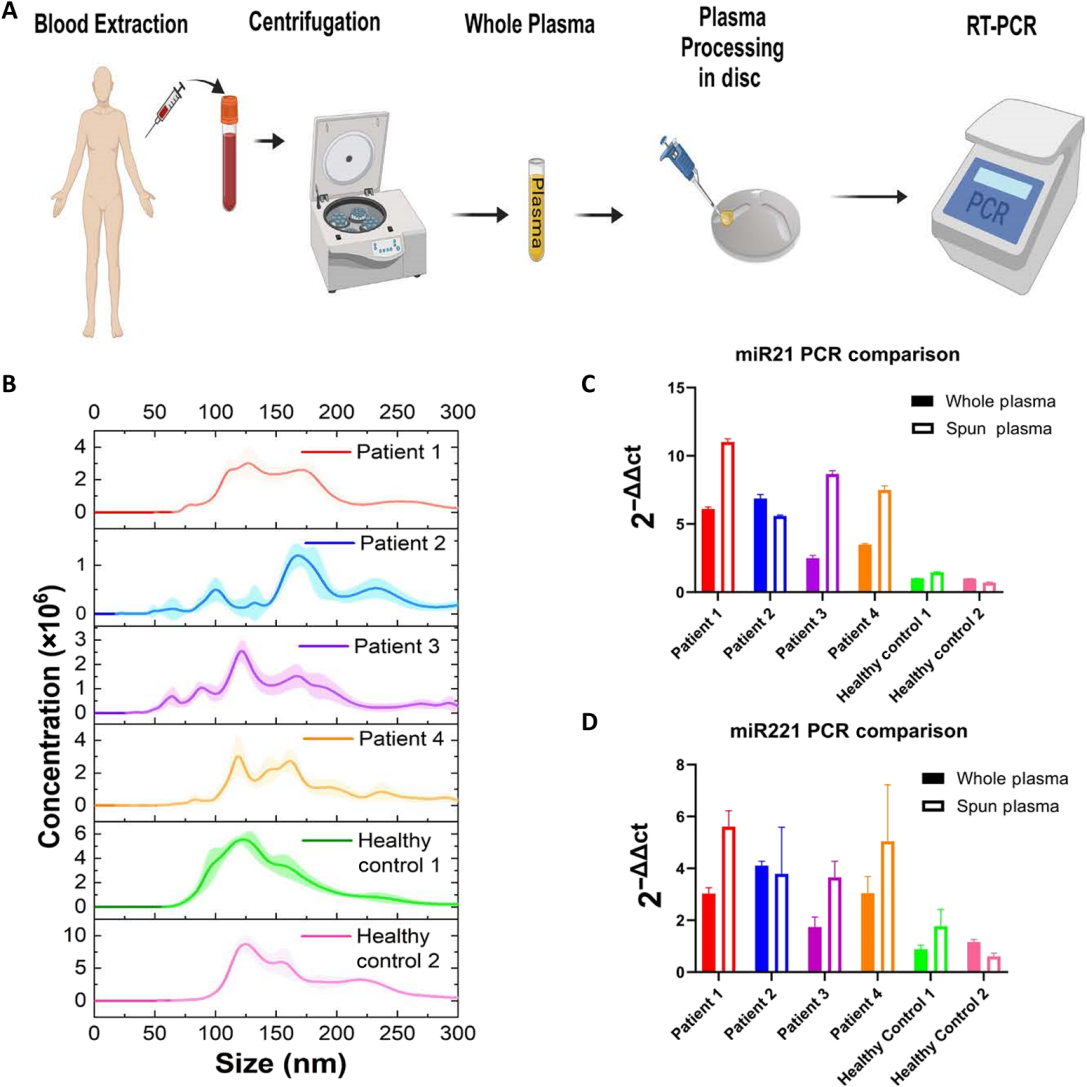
Demonstration of biomarker enrichment for CRC patient samples using the ASCENDx platform. (**A**) Schematic showing sample-to-answer workflow for PCR analysis. (**B**) NTA results for patient samples after disc spinning. The dashed line indicates that particles below ~75 nm are cut off. (**C** and **D**) PCR results showing the relative expression levels of miR21 and miR221 in whole plasma (solid bars) and plasma processed through our device (hollow bars), respectively. Our ASCENDx platform adds significant biomarker enrichment, as evidenced by the amplified signal between the spun sample and whole plasma. (A) was made with BioRender.com.

To highlight the diagnostic potential of our ASCENDx platform through traditional RT-PCR analysis of CRC patient samples, we analyzed six human plasma samples (four CRC patient samples and two healthy donors). The samples that were positive for CRC are denoted patient 1, patient 2, patient 3, and patient 4, while the control samples are denoted healthy control 1 and healthy control 2. Figure 4A illustrates the entire sample-to-answer process, where patient blood samples were first extracted and conventionally centrifuged to isolate the plasma. This plasma sample is then loaded onto our acoutstofluidic disc, spun, and tested through RT-PCR. Figure 4B shows the relative size distribution of each plasma sample after spinning in our ASCENDx device. The relative expression profiles of miR21 and miR221 were measured in each sample, as shown in Fig. 4 (C and D), showing high specificity between patients with cancer and healthy individuals. Furthermore, to investigate the effects of our enrichment platform on diagnostic outcomes, we conducted RT-PCR of unprocessed plasma and plasma processed through our ASCENDx device for each patient sample. For both CRC miRNA biomarkers, our results indicate that samples processed using the ASCENDx device have greater diagnostic utility than unprocessed plasma. Pearson correlation values between patient-averaged 2^−∆∆Ct^ values for each miRNA and diagnosis noticeably increased as a result of ASCENDx processing, from 0.764 to 0.900 in the case of miR-21 and from 0.802 to 0.902 in the case of miR-221. These findings agree with trends seen in the analysis of exosomes of patients with CRC (*78*) and further validate the utility that our exosome enrichment platform provides for improved biomarker detection.

### ASCENDx: Sample to answer

**Fig. 5.**
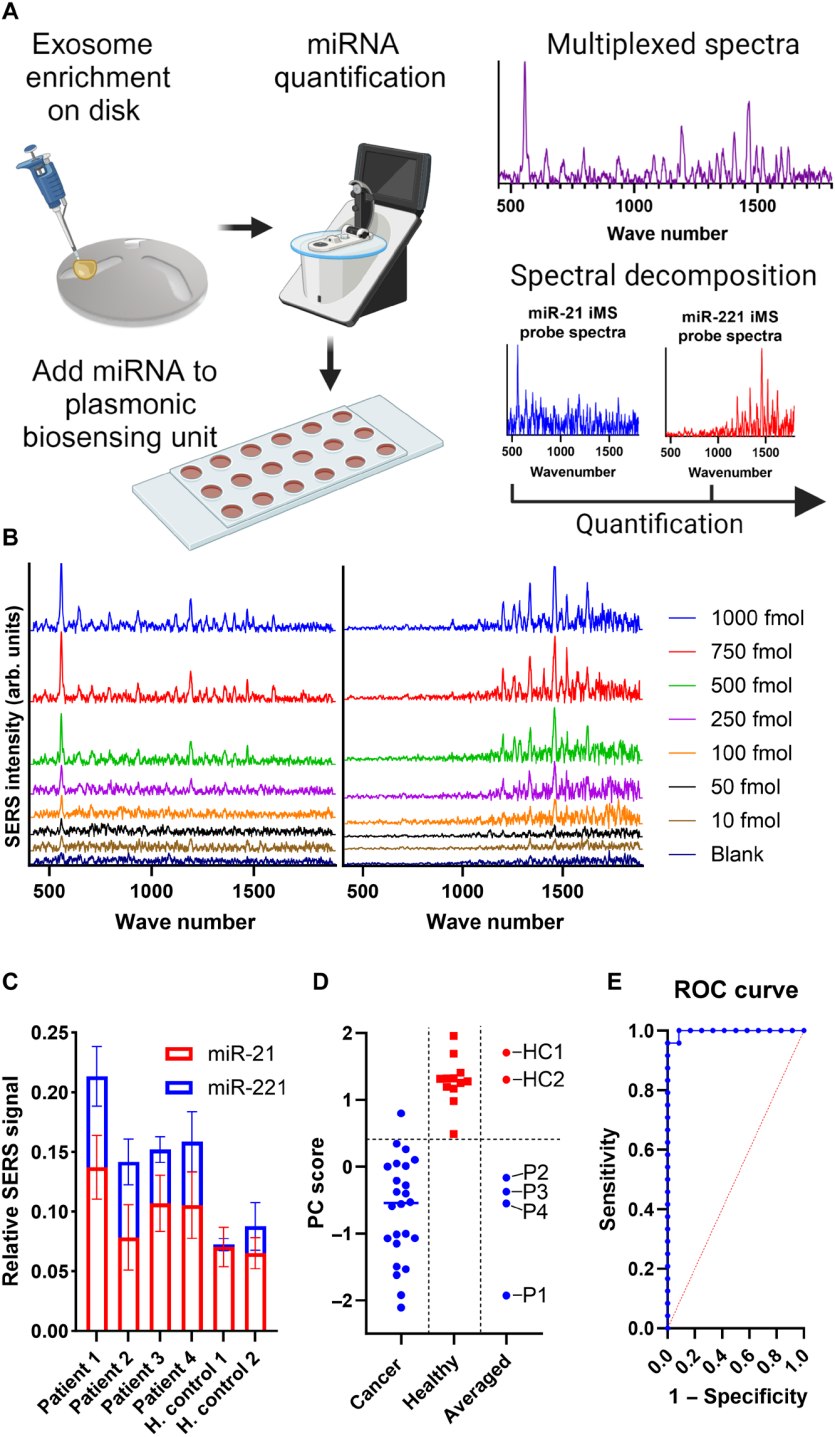
Direct sensing of miRNA biomarkers for the detection of CRC using the ASCENDx platform. (**A**) (Left) Scheme depicting patient sample workflow for biomarker detection using the ASCENDx platform. (Right) Representative multiplexed SERS spectra composed of individual iMS probe signals. (**B**) Representative spectra depicting miR-21 iMS probe and miR-221 iMS probe signal in response to the addition of synthetic target. (**C**) Relative SERS signal increases for each iMS probe for each patient sample analyzed, *N* = 6 per patient. (**D**) Principal components analysis results of all samples analyzed, *N* = 24 for the cancer group, and *N* = 12 for the healthy group. (**E**) ROC curve generated using the relative SERS signal increase for each iMS probe after spectral unmixing for each recorded spectra, *N* = 24 for the cancer group, and *N* = 12 for the healthy group. (A) was made with BioRender.com.

Last, we demonstrated the diagnostic utility of the ASCENDx platform through multiplexed detection of circulating CRC biomarkers from patient plasma samples. The left side of Fig. 5A depicts the sample workflow: Enriched exosomal RNA from the acoustofluidic disc of ASCENDx is quantified and added to the nanostar-functionalized plasmonic biosensing unit integrated with iMS probes. The miRNA biomarkers miR-21 and miR-221 were chosen as targets because there is evidence that both sequences are significantly up-regulated in patient plasma (*79*–*81*). The right side of Fig. 5A illustrates the process by which the multiplexed SERS spectra resulting from ASCENDx analysis undergo spectral unmixing for individual probe signal quantification. The oligonucleotide SH-C6-AAAAA-CY5 was used to test the repeatability and reproducibility of the SERS signal generated from the nanostar substrate. The spectra of 20 distinct sensing regions were recorded and analyzed, with representative raw spectra shown in fig. S10A. This resulted in an average peak height at 557 cm^−1^ after signal normalization of 0.940 ± 0.033 (3.5%), shown in fig. S10B. Next, to demonstrate how the assay can be repeatable and reproducibly prepared, the SERS signal of 10 different sensing regions was recorded for each iMS probe at different stages throughout the assay preparation process. The normalized peak height at 557 cm^−1^ for the miR-21 probe and at 1461 cm^−1^ for the miR-221 probe in the “ON” and “OFF” configurations is shown in fig. S10C. After signal normalization, the average peak height for the miR-21 iMS probe in the ON configuration was 0.965 ± 0.024 (2.5%) and 0.109 ± 0.007 (6.4%) for the OFF configuration. The average peak height after normalization for the miR-221 iMS probe in the on configuration was 0.969 ± 0.018 (1.8%) and 0.141 ± 0.014 (9.9%) in the OFF configuration.

Figure 5B shows representative changes in SERS intensity as a function of synthetic analyte added to the sensing region of the substrate for miR-21 and miR-221 iMS probes. Both iMS probes display highly linear behavior across the range of targets investigated, with *R*^2^ values of 0.9905 and 0.9748 for miR-21 and miR-221 when measured in triplicate. The limit of detection for miR-21 is 19 pg and that for miR-221 is 17 pg. Twenty-nanogram aliquots of enriched small RNA obtained from the acoustically enriched patient samples were added to individual sensing regions of the plasmonic nanostar-based biosensing unit, which was integrated with both miR-21 and miR-221 iMS probes. Each patient sample was analyzed six times, and the signal contributions for each iMS probe are shown in Fig. 5C. The samples positive for CRC had greater iMS probe signal contributions for both miRNA biomarkers compared to the control group.

To confirm the specificity of the ASCENDx analysis, the Pearson correlation coefficient between the mean 2^−∆∆Ct^ values of each miRNA of interest and the corresponding iMS probe signal increase for each patient was analyzed, resulting in a Pearson correlation coefficient of 0.75 and 0.67 for miR-21 and miR-221, respectively. To assess the diagnostic performance of the ASCENDx analysis, the relative iMS probe signal increases for each patient were analyzed via principal components analysis, with results shown in Fig. 5D. By establishing a threshold principal component value at 0.4, 35 of 36 samples were completely partitioned by health status, resulting in a test with 95.8% sensitivity and 100% specificity. The iMS probe signal contributions for all samples were averaged per patient and were completely partitioned on the basis of health status upon reanalysis. Receiver operating characteristic (ROC) curve analysis of all patient data is shown in Fig. 5E, resulting in an area under the curve of 0.997 [0.985, 1.00] with 95.8% sensitivity and 91.7% specificity at a classification cutoff at 0.5. Adjusting the classification cutoff to 0.8 results in a diagnostic assay performance of 95.8% sensitivity and 100% specificity.

## DISCUSSION

Circulating exosomal cargo in the form of oligonucleotides and proteins can provide insight into patient health status, including disease development and progression. However, the rate of biological research and clinical translation of exosomal cargo profiling is hindered by the difficulty of removing nonexosomal contaminants and concentrating the biomarkers of interest. Moreover, exosomes are often isolated and analyzed separately, further adding to the complexity and processing time required. The ASCENDx liquid biopsy analysis platform introduced in this work addresses the shortcomings associated with traditional exosomal processing and subsequent miRNA detection.

The end-to-end patient sample analysis ASCENDx platform provides exosome enrichment, on-disc label-free SERS verification of exosome enrichment, and the amplification-free multiplexed detection of miRNA biomarkers contained within that enriched sample. The miniature acoustofluidic disc is capable of isolating exosomes with high yield and purity from low-volume biofluid samples by applying SAWs coupled into a fluid droplet on which a disc is placed (table S1). Plasmonic nanostar particles were optimized and integrated onto the surface of the acoustofluidic disc to facilitate label-free SERS verification of exosome enrichment after acoustic separation. RT-PCR results confirmed that the miRNA content of enriched exosome samples was more strongly correlated with health status compared to untreated plasma, providing a compelling example of how the ASCENDx technology can readily be adapted for biomarker discovery applications. The plasmonic biosensing unit of the ASCENDx platform effectively discriminated between acoustically enriched miRNA derived from CRC-positive patients and healthy controls with 95.8% sensitivity and 100% specificity, a rapid sample-to-answer time of 30 min, and was strongly correlated with RT-PCR results. Moreover, to the best of our knowledge, this is the first report of multiplexed, amplification-free direct detection of miRNA biomarkers from acoustically enriched patient plasma samples. The ASCENDx workflow established here acts as a proof-of-concept validation and demonstrates the synergy between these sample processing and biosensing platforms.

The ASCENDx workflow can be applied to various health status assessment applications because of the inherently versatile underlying technologies. Regardless of the exosomal biomarker of interest, all exosome-containing biofluids are eligible for enrichment. Easily accessible samples such as saliva are strong candidates for biomarker separation in future studies. While CRC was used to demonstrate the diagnostic advantage provided by the ASCENDx workflow, ailments ranging from several types of cancer, neurodegenerative diseases, and even acute injuries that cause circulating miRNA dysregulation are candidates for investigation. Last, the flexibility provided by the ASCENDx technology allows for the rapid design, validation, and integration of probes for any miRNA biomarker target. Future development of the platform requires careful consideration of the transducer design, location, and frequency as well as the droplet volume and viscosity to fully optimize disc rotation. We intend to explore and optimize such aspects of ASCENDx as we develop the platform further and seek unexplored applications.

In summary, we have established our ASCENDx for rapid and effective sample enrichment and subsequent miRNA biomarker detection. This technology will significantly simplify analytical protocols and help unlock exosome’s transformative potential in biomedical research and diagnostic applications.

## MATERIALS AND METHODS

### Device fabrication

The SPFTs were fabricated by depositing a 5-nm-thick layer of Cr and a 150-nm-thick layer of Au onto a 128° Y-cut LiNbO3 wafer (Precision Micro-Optics, USA) using electron beam evaporation. The photoresist patterns on the LiNbO3 wafer used for the metal evaporation were fabricated via photolithography, and the excess metal was removed using a standard lift-off process with acetone. All SPFTs were composed of 116 pairs of electrode fingers. Each SPFT resulted in an SAW frequency of 30 MHz. Using silver epoxy, external wires were bonded to the electrodes (MG Chemicals, USA). A biopsy punch created the PDMS ring that confines the droplet. The PDMS ring and LiNbO3 substrate were treated with an oxygen plasma to promote surface bonding, followed by a post-bake at 65°C for 8 hours. A function generator (DG 3012C, Tektronix, USA) and an amplifier (25A250A, Amplifier Research, USA) activated the SPFTs and generated SAWs. The PDMS discs were fabricated using standard SU-8 soft lithography and the PDMS mold-replica process. Discs can be left open or covered by a thin membrane to prevent sample evaporation or ambient contamination. All experiments in this work were performed using an enclosed disc.

### Image acquisition and analysis

The images were acquired using an upright microscope (BX51WI, Olympus, Japan) and a charge-coupled device camera (CoolSNAP HQ2, Photometrics, USA). The disc’s rotational speed was measured using a smartphone camera with 30 to 240 frames per second. The data and figures were analyzed using ImageJ (National Institutes of Health, USA). The nanoparticle size distribution was analyzed using an NS500 running NTA software 3.4 by Malvern Panalytical.

### Protein quantification

Proteins were quantified using a bicinchoninic acid (BCA) protein concentration assay kit (Thermo Fisher Scientific, Rockford IL, USA). To establish a quantification curve, 10 μl of bovine serum albumin solution (0, 0.025, 0.075, 0.125, 0.250, 0.500, 0.750, and 1.000 mg/ml) was added to the wells of a microtiter plate. Then, 200 μl of BCA working solution was added to the wells and incubated at 37°C for 30 min. Absorbance was measured using a BioTek Synergy H1 Multimode Reader (Biotek, Winnoski VT, USA).

### Synthesis of bimetallic nanostars

To prepare 100 ml of bimetallic nanostar solution, 1 ml of 10 nM 12-nm gold seed solution was added to 100 ml of 25 mM HAuCl4 and 100 μl of HCl. Next, 500 μl of 6 mM AgNO3 was added to the mixture, followed rapidly by 500 μl of 0.1 M ascorbic acid and then between 150 and 1000 μl of 0.1 M AgNO3 depending on the type of bimetallic nanostar. One hundred microliters of 30% ammonia solution was then added to terminate the reaction (*74*).

### Substrate fabrication

Bimetallic nanostar substrates were prepared using 8-mm-diameter coverslips in the case of “on-disc” SERS measurements, and 25 mm–by–75 mm microscope slides in the case of the miRNA assay. All substrates were cleaned using piranha solution for 1 hour, washed in deionized (DI) water, and dried overnight at 110°C. Substrates were then placed into sealed vapor deposition chambers with 50 μl of *N*-(2-aminoethyl)-3-aminopropyltriethoxysilane and left in an oven for 48 hours at 90°C. Once vapor deposition was complete, substrates were washed in toluene, ethanol, and DI water to remove noncovalently linked aminosilane on the substrate surface. Substrates were then submerged into a bimetallic nanostar solution for 3 days, at which point they were removed, rinsed with DI water, and stored under nitrogen until further use (*82*).

### COMSOL simulation of bimetallic nanostars

The wave optics package in COMSOL Multiphysics 6.0 simulates the magnitude of local electric field enhancement achieved by different morphologies of bimetallic nanostar particles. The optical parameters described by Rakic *et al.* (*83*) were used for gold and silver in this model. The surroundings were modeled as water, using the optical parameters described by Daimon and Masamura (*84*). The entire model was meshed using the extremely fine setting defined by the predetermined conditions in the wave optics module.

### SERS measurements on the disc of ASCENDx

Measurements were recorded using a Horiba Jobin Yvon LabRam ARAMIS system. The excitation wavelength used for this study was 633 nm at 50% power. Spectra were recorded for 10 s and three accumulations each. All spectra were recorded using a 100× objective.

### Collection of patient samples

Plasma samples were stored at −80°C until testing. Samples were processed as part of standard care for patients with excess plasma not needed for testing stored frozen. Patient plasma samples were sourced from the Southern Division of the Cooperative Human Tissue Network at Duke University, which has Institutional Review Board (IRB) approval to procure leftover clinical plasma samples from Duke patients and distribute them anonymously to Duke University investigators under IRB exemption (approval number Pro00075497). A Peltier cooling plate (TEC1-12730, Hebei IT, China) was placed under the device during blood and plasma separation experiments to reduce heating and maintain sample integrity.

### miRNA isolation from patient samples

miRNA was isolated from patient plasma or spun samples using the commercially available mirVANA PARIS kit (Invitrogen), using the manufacturer’s protocol. For each sample, 250 μl was added to an equal volume of 2× denaturing solution, followed by 500 μl of acid phenol. After homogenization, samples were centrifuged at 11,000*g* for 5 min. The entire aqueous phase was then collected and mixed with one-third volume of ethanol and added to a tube containing a glass fiber filter. The sample was then passed through the filter, and the flow through was collected. To enrich the sample for small RNAs, two-thirds volume of ethanol was added to the flow through and passed through a fresh glass fiber filter. This filter was then washed with the included miRNA washing buffers as indicated by the manufacturer’s protocol. The enriched small RNA was eluted with 50 μl of DI water. Nucleotide quantification was done using a Nanodrop OneC (Thermo Fisher Scientific).

### cDNA synthesis

The isolated small RNA from each patient was used to synthesize cDNA for future RT-PCR analysis. cDNA was prepared using the miRCURY LNA RT kit (Qiagen), following all manufacturer protocols. A reverse transcriptase master mix was prepared with 5× miRCURY RT SYBR Green Reaction Buffer, 10× miRCURY RT Enzyme Mix, UniSP6 RNA spike-in control, and ribonuclease (RNase)–free water. Isolated small RNA (10 ng) was added to each RT reaction. All RT reactions were incubated for 60 min at 42°C and then at 95°C for 5 min. RT reaction mixtures were then immediately cooled to 4°C until further use.

### Substrate assembly

To fabricate the well system onto the surface of the nanostar substrate, a Cricut cutting machine was used to cut double-sided adhesive from 3M into squares with an edge length of 7 mm, and with a hole 4 mm in diameter removed from the center. This adhesive was used to mount PDMS wells with the same dimensions and 4 mm in height onto the surface of the substrate.

### iMS probe oligonucleotide sequences

The sequence of the miR-21 iMS probes, placeholder, and synthetic target used in this study was 5′-SH-AAAAAGTCTGTATTAAAAAA-TAGCTTATCAGAC-Cy5-3′, 5′-TCAACATCAGTCTGATAAGCT-ATTTT-3′, and 5′-TAGCTTATCAGACTGATGTTGA-3′, respectively. The sequence of the miR-221 iMS probes, placeholder, and synthetic target used in this study was 5′-SH-AAAAAGCAGAATTAAAAAAA-AAAGCTACATTGTCTGC-cy3-3′, 5′-GAAACCCAGCAGACAAT-GTAGCTTTTT-3′, and 5′-AGCTACATTGTCTGCTGGGTTTC-3′, respectively.

### RT-PCR

RT-PCR was performed using the miRCURY LNA SYBR Green PCR Kit (QIAGEN) and miRCURY LNA miRNA PCR Assays (QIAGEN). The PCR assays used in this study were designed to detect hsa-miR-21-5p, hsa-miR-221-3p, and the UniSp6 spike-in control. cDNA templates from the RT reactions were all diluted at 1:30 before their addition. A master mix containing 2× miRCURY SYBR Green Master Mix, 1 μl of PCR primer mix, and 1 μl of RNase-free water was prepared for each of the three targets of interest. Each target was run in triplicate for each patient sample using a Roche LightCycler 480 real-time cycler. Run conditions were set according to the miRCURY LNA miRNA PCR Assays Quick-Start Protocol. Relative expression was evaluated via the 2(–∆∆Ct) method. All samples were analyzed in triplicate.

### iMS assay preparation

The iMS assay for both miR21 and miR221 iMS probes, 1 μM probe in 1× phosphate-buffered saline (PBS) and 0.01% Tween 20, and 100 μM tris(2-carboxyethyl)phosphine (TCEP) was incubated for 3 hours. In each substrate well, 3 μl of iMS probe TCEP solution and 4.5 μl of 100 μM mercaptohexanol (MCH) solution were diluted in 1× PBS and 0.02% Tween 20, 12 μl of 1× PBS and 0.02% Tween 20, and 15 μl of 20× PBS. After loading, the substrate was placed on a shaker overnight. All wells were then washed using 1× PBS and 0.01% Tween 20 solution. Next, 30 μl of 100 μM MCH was added to each well, and the substrate was placed in a heated shaker set to 35°C for 30 min. All wells were then washed with 1× PBS and 0.01% Tween 20 solution. After washing, 30 μl of 3 μM placeholder solution was added to each well and placed into a heated shaker for 1 hour at 45°C. Substrate wells were then washed for a final time, and blank signals were then recorded for all wells to ensure that the iMS assay was correctly prepared in all wells. For each patient, 20 ng of total small RNA was added to a PBS and Tween 20 mixture, resulting in patient sample solution containing a total of 20 ng of small RNA in 1× PBS and 0.01% Tween 20 with a total volume of 10 μl. Patient sample solutions were added to the prepared substrate wells and allowed to react for 30 min. All spectra were recorded from regions of the substrate with no prior laser exposure.

### SERS signal collection and analysis

All iMS assay measurements were performed using a Renishaw InVia confocal Raman microscope equipped with a 632.8-nm HeNe laser and a 10× objective. Before each measurement, the laser spot was focused onto the surface of the substrate to ensure that the same amount of substrate area was exposed to the laser during every measurement. Using ImageJ, the area of this laser spot was measured and used to calculate the absolute mass of the target within the sensing region.

All recorded spectra were processed in MATLAB, where they were background subtracted and smoothed with a Savitzky–Golay filter (five-point window and first-order polynomial). For the analysis of multiplexed spectra, the spectral decomposition developed by Lutz *et al.* (*85*) was used to determine to what amount each individual iMS probe signal contributed to the recorded spectra. This method uses reference spectra for the individual miRNA probes, the multiplexed blank signal of each well before the addition of the patient sample solution, and the multiplexed signal after the addition of patient sample. After removing the signal contribution from the multiplexed blank spectra, the MATLAB functions lsqnonnneg and fmincon, which solve nonnegative least-squares curve fitting problems and minimize a system of nonlinear equations, respectively, are used to determine the contribution of each reference spectra to the patient sample signal. This analysis generates minimally constrained coefficients by which the reference spectra are multiplied to produce the best fit to the input patient spectra. These coefficients then allow for the direct comparison between patient spectra for each individual iMS probe signal.

## Supplementary Materials

### This PDF file includes:

Note S1 Figs. S1 to S10 Table S1 Legends for movies S1 and S2
