# Supplementary material for: Acoustic separation and concentration of exosomes for nucleotide detection: ASCENDx
Source: Sci Adv. 2024 Mar 8;10(10):eadm8597. doi: 10.1126/sciadv.adm8597 (PMC10923504; doi:10.1126/sciadv.adm8597)
Supplement: Supplementary file 1 — Note S1 Figs. S1 to S10 Table S1 Legends for movies S1 and S2 [file sciadv.adm8597_sm.pdf]

Science Advances
AAAS

Supplementary Materials for

Acoustic separation and concentration of exosomes for nucleotide detection: ASCENDx

Ty D. Naquin *et al.*

Corresponding author: Luke P. Lee, lplee@bwh.harvard.edu; Tuan Vo-Dinh, tuan.vodinh@duke.edu; Tony Jun Huang, tony.huang@duke.edu

Sci. Adv. **10**, eadm8597 (2024)
DOI: 10.1126/sciadv.adm8597

The PDF file includes:

Note S1
Figs. S1 to S10
Table S1
Legends for movies S1 and S2

Other Supplementary Material for this manuscript includes the following:

Movies S1 and S2

Note S1: Acoustic simulations

The simulation of the fluid streaming velocity within a droplet was conducted in COMSOL MULTIPHYSICS 5.6 with a fluid mechanical module. To simulate the acoustic streaming velocity distribution and magnitude within a droplet, a body force was applied to one-half of a 3D water droplet with a diameter of 4 mm and a height of 1.06 mm. The body force can be expressed as:

$$FF_{zz} = -\rho\rho(1+\alpha\alpha^{2})AA^{2}\omega\omega^{2}kk_{ii}\alpha\alpha\alpha\alpha^{2(kk_{ii}xx+2kk_{ii}zz)},$$
$$FF_{xx} = -\rho\rho(1+\alpha\alpha^{2})AA^{2}\omega\omega^{2}kk_{ii}\alpha\alpha^{2(kk_{ii}xx+2kk_{ii}zz)},$$

where ρ is the water density, $\alpha = 2.47$ is the attenuation coefficient, $A = 1$ nm is the amplitude, ω is the angle frequency, $k_i = \omega/c_l$ is the imaginary part of the wave number, and $c_l = 3931+i*68.1$ m/s is the wave speed of the leaky surface acoustic wave in water. The fluid pressure on a random point at the bottom of the droplet is set to 0 Pa to help the computation converge faster. The model was calculated with a stationary solver. The bottom of the droplet was set as a non-slip condition, and the open edges of the droplet were set as slip conditions. A cross-section 100 µm above the bottom of the 3D droplet simulation, as seen in Supplemental Figure 1, was shown in Figure 2b. The simulation results shown here model a device with a single SPFT pair rather than the two SPFT pairs used in our experimental results in Figure 2.

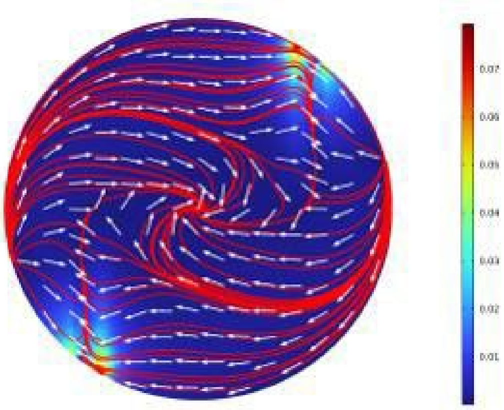

Fig. S1. Numerical simulation results investigating fluid streaming velocity within the water droplet.

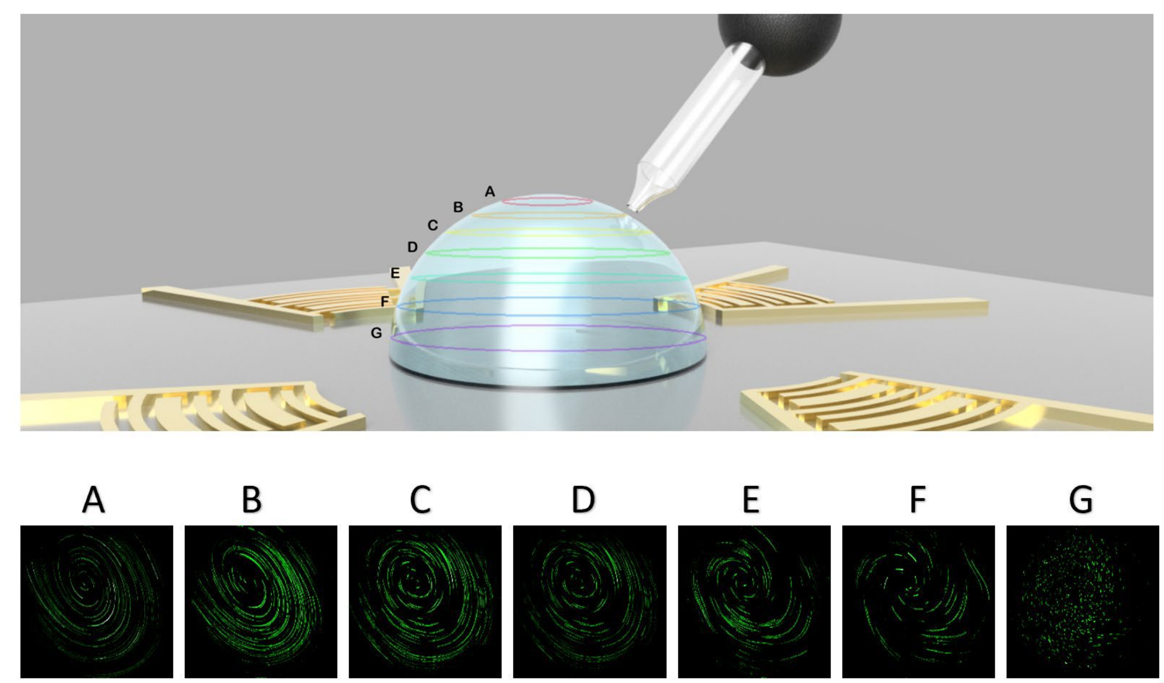

Fig. S2. Experimental acoustic streaming pattern within the droplet. (A-G), A series of images showcasing the streaming pattern at different layers of the droplet. As the particles spiral down to the bottom of the droplet, the streaming pattern acts as a whirlpool that drives particles to aggregate at the center.

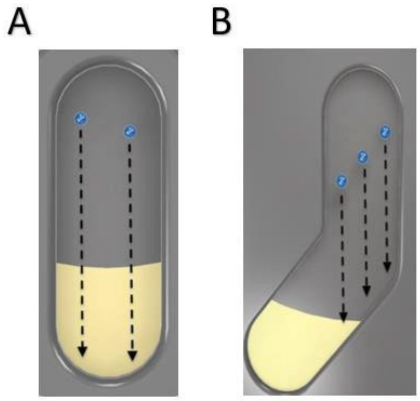

Fig. S3. Tilted channel design of the ASCENDx disc. (A), Schematic showcasing the distance traveled by particles in a straight channel. **(B),** Shortened path length experienced by particles in a tilted channel.

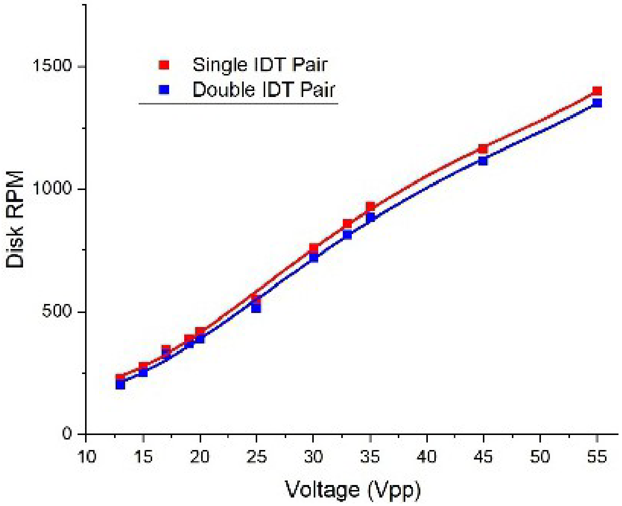

Figure S4. Disc RPM for one and two IDT pairs.

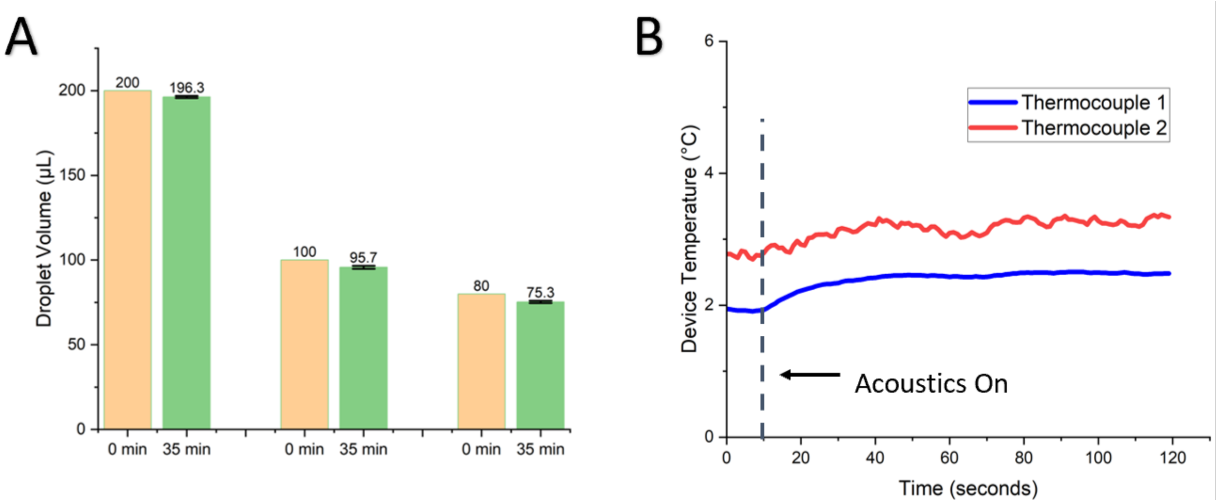

Fig. S5. **Characterization of droplet temperature and evaporation.** **(A)** Droplet volume before and after 35 minutes of applied acoustics. **(B)** Droplet temperature before and during acoustic actuation.

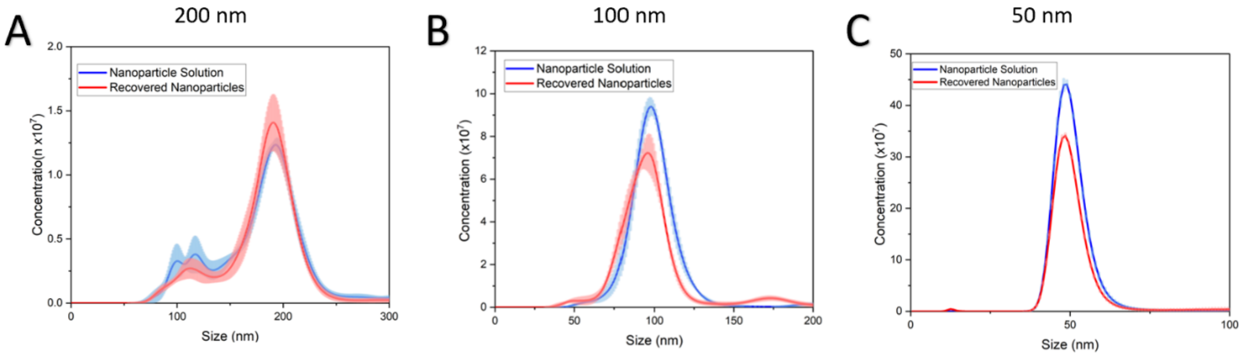

Figure S6: Nanoparticle tracking analysis of particle enrichment. (A-C) NTA concentration results before and after ASCENDx enrichment for 200 nm (A), 100 nm (B), and 50 nm (C) particles.

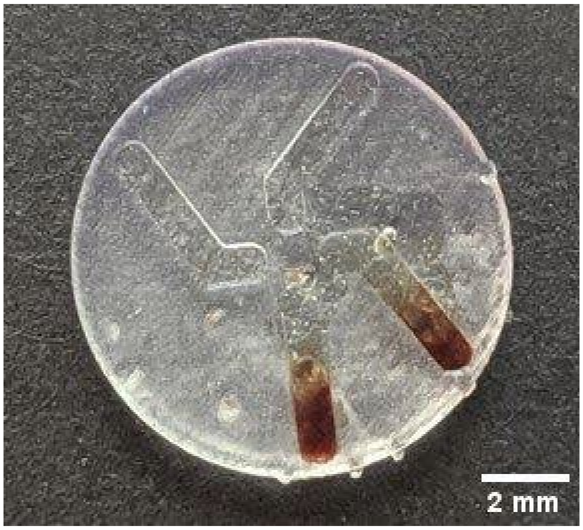

Fig. S7. Separation of plasma from blood in straight and bent channels of the ASCENDx device.

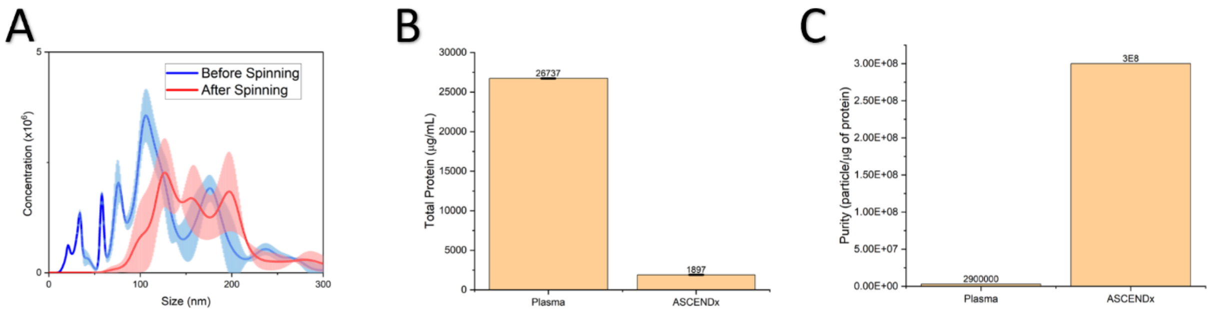

Figure S8: Yield and purity of the ASCENDx platform. (A) Particle concentration and distribution before (blue) and after (red) ASCENDx processing. **(B)** Protein concentration in plasma before and after enrichment with ASCENDx. **(C)** Purity from protein contaminants of ASCENDx enriched exosome samples.

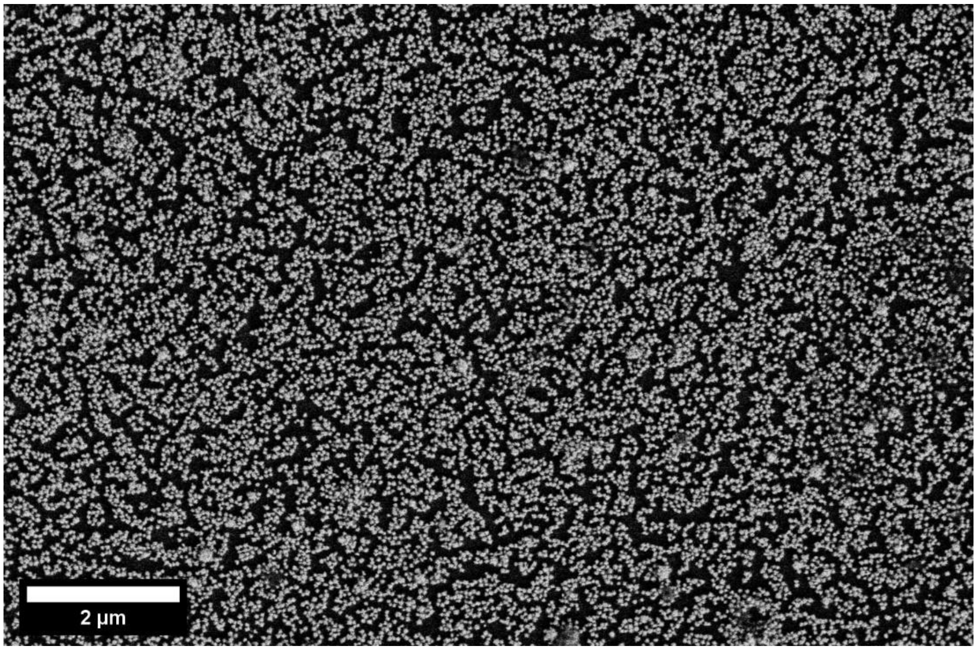

Fig. S9. SEM image of nanostars functionalized onto the ends of the acoustofluidic spinning disc.

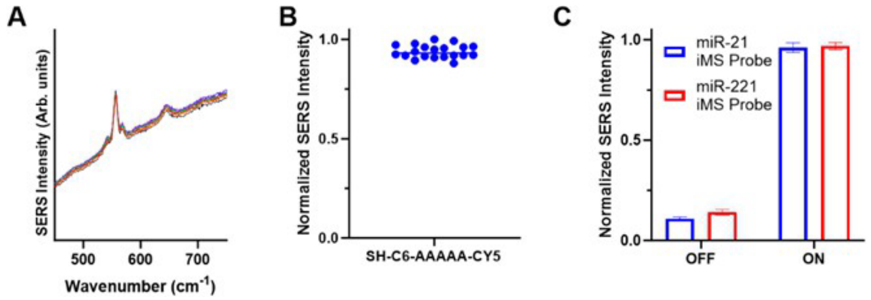

Figure S10. Plasmonic biosensing unit characterization. (A) Representative spectra of SH-C6-AAAAA-CY5 recorded from different sensing regions, N=8. **(B)** The normalized peak height at 557 cm^{-1} of SH-C6-AAAAA-CY5 SERS spectra recorded from different sensing regions, N=20. **(C)** Normalized SERS intensities of each iMS probe in the "ON" and "OFF" configurations, N=10.

Parameter	Differential Ultracentrifugation	Polymer precipitation	Membrane Affinity	Immunoaffinity Capture Beads	ASCENDx
**Yield (%)**	5-25%	~40%	~9%	~30%	**~86%**
**Purity (particles/µg of protein)**	$3\times10^5 – 4\times10^7$	$6.1\times10^7–2\times10^8$	~1x10^7	~2×10^6	**~3×10^8**
**Processing Time**	8 – 12 hours	~30 min – overnight incubation	~30 minutes	~6-24 hours	**~30 minutes**
**Minimal Processing Volume**	~5 mL	~1 mL	~200 µL	~500 µL	**~3 µL**

Supplementary Table 1: Competitive analysis for different exosome isolation methods.

Movie S1: Top view of acoustofluidic disc rotation of the ASCENDx device.

Movie S2: Side view of acoustofluidic disc rotation of the ASCENDx device.
